# Stone in the distal jejunum presenting as small bowel obstruction: A case report

**DOI:** 10.1016/j.ijscr.2018.09.045

**Published:** 2018-10-04

**Authors:** Nafeesah Fatimah, Abubaker Shafiq Ahmed, Muhammad Umar Warraich, Usman Ismat Butt, Qamar Ashfaq Ahmad, Mahmood Ayyaz

**Affiliations:** Department of General Surgery, Services Institute of Medical Sciences, Lahore, 54600, Pakistan

**Keywords:** Gallstones, Intestinal obstruction, Fistula repair

## Abstract

**Introduction:**

Gallstone ileus is a rare presentation of cholelithiasis accounting for around 4% of cases of small bowel obstruction under 65 years of age but rises to 25% in those more than 65 years of age. Most gall stones, if impact in the bowel, are seen to lodge at the ileocecal valve as this is the most narrow part of the bowel.

**Presentation of case:**

We present a case of small intestinal obstruction because of a gallstone stuck in the distal jejunum. Patient was explored and one-stage surgery was done i.e. cholecystectomy, enterotomy and stone retrieval, and fistula repair. Our patient had uneventful postoperative recovery and was discharged after a week.

**Discussion:**

Gallstone ileus is a rare entity presenting a significant diagnostic challenge to surgeons due to lack of specific findings. Moreover, the surgical management options to be adopted are diverse and depend upon the condition of the patient. Stable patients can be managed with two-stage procedures i.e. enterotomy and stone retrieval earlier with cholecystectomy in an elective setting. Our patient was managed with one-stage procedure i.e. cholecystectomy was done in the same setting and our patient did well.

**Conclusion:**

Gallstone ileus needs to be kept in the differential diagnosis list of intestinal obstruction in middle aged patients even in the absence of history of gallstones as this may be the first presentation of gallstone disease.

## Highlights

• Gallstone impaction in the distal jejunum is rare.
• Pre-operative diagnosis is a challenge as findings remain equivocal on imaging studies.
• One or two stage procedure can be performed to alleviate obstruction.

## 1 Introduction

Gallstones can present in various ways in both outpatient and emergency department, with gallstone ileus being one of the rare presentations. Gallstone ileus occurs due to a gallstone impacted in the gastrointestinal tract through a biliary-enteric fistula with most common site of impaction being the distal ileum, however, stones impacted in gastric outlet i.e. Bouveret’s syndrome have also been reported [1]. These complications are usually seen in elderly females with multiple co-morbidities [2]. We present a rare case of gallstone impaction in the proximal ileum leading to bowel obstruction in a male patient who was managed with exploration and had uneventful postoperative course. The work has been reported in line with the SCARE criteria [3].

## 2 Presentation of case

A hypertensive 58 year old male presented to us in surgical emergency with a 3 days history of abdominal pain, vomiting and absolute constipation. He has never had experienced such an abdominal pain before. There were no other co-morbidities and patient denied any history of smoking, alcohol abuse, or use of illicit drug. On examination, patient was hypotensive, and tachycardiac without jaundice. Abdominal examination showed distended abdomen with guarding in the periumbilical region and right hypochondrium with sluggish bowel sounds. Digital rectal examination showed collapsed rectum.

**Fig. 1:**
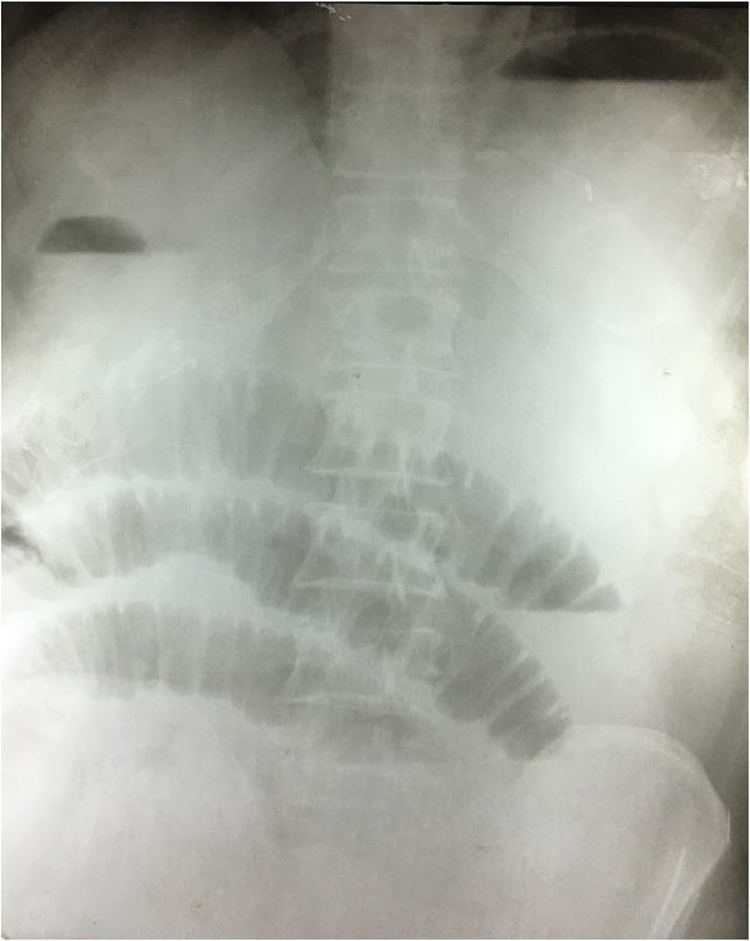
Erect abdominal X ray showing air-fluid levels and dilated bowel loops.

Investigations showed Hb of 15.4 g/dl with neutrophilia (1,5000/μm) and serum creatinine of 3.9 mg/dl. No electrolyte imbalance was noted and serum amylase and lipase levels were within normal limits. Chest X ray was normal while abdominal x ray showed dilated gut loops (Fig. 1). Ultrasound scan reported multiple stones in the gall bladder along with excessive bowel gas shadows. CT Abdomen was planned but patient refused to undergo CT Scan. Hence, it was decided to explore the patient.

**Fig. 2:**
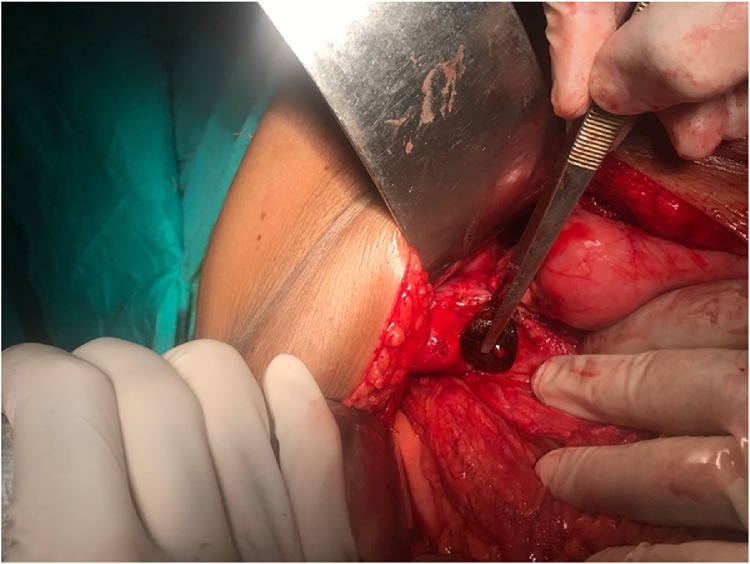
Stone being retrieved from the jejunum.

After nasogastric decompression and fluid resuscitation, patient was shifted to the theatre four hours after admission in surgical emergency. Patient was operated via midline umbilical saving incision. Laparotomy findings were a cholecystoduodenal fistula along with dense adhesions of gall bladder with adjacent structures and a 5 × 4 cm stone was stuck three and a half feet from duodenojejunal flexure in the distal jejunum (Fig. 2). Enterotomy was done and bowel was closed in transverse fashion. Since the cholecystoduodenal fistula was noted along with purulent discharge around the area of fistula, it was decided to do a one-stage procedure. Hence, cholecystectomy was done and the duodenum was repaired. To provide duodenal rest, pyloric exclusion was done and jejunual loop was anastomosed to the posterior surface of the stomach in an isoperistaltic retrocolic fashion to establish bowel continuity. An additional note was made of a paraumbilical hernia containing omentum and the defect was repaired. Pelvic and subhepatic drains were placed. The nasogastric tube was kept in-situ. Patient was shifted to surgical ward postoperatively. He remained well in the postoperative period, nasogastric tube was removed on the second post-op day and oral feed was started after removal of the nasogastric tube, while drains were removed on the third postop day. His serum creatinine levels fell gradually to 1.5 mg/dl over the next five days indicating that he had pre-renal azotemia due to dehydration secondary to small bowel obstruction. Patient was discharged on the 7th postoperative day.

## 3 Discussion

Gallstone ileus is a relatively rare entity in surgical clinics with incidence ranging from 30 to 35 cases/100,000 admissions [4]. It has been shown that 0.3% −0.5% patients with gallstones develop such a complication but 25% of such non-strangulated mechanical bowel obstruction were seen in geriatric age group with female to male ratio of 3.5–6.0:1.0 [1,4]. Despite continuing medical advances, misdiagnosis is common and mortality is high.

Gallstone ileus is usually seen in patients with recurrent attacks of acute calculus cholecystitis harbouring larger gallstones [5]. The most common fistulas are cholecystoduodenal, while cholecystocolic and cholecystogastric fistulas have also been reported [1,2,4]. The level of obstruction is dependent upon the size of the gallstone. Stones ≤2.5 cm is size usually pass spontaneously while stones >2.5 cm in size are obstructed mostly at the site of ileocecal valve hence there seems to be a possible role of nasogastric decompression and fluid resuscitation in the management of gallstone ileus [4,7]. However, stones obstructing the sigmoid colon, proximal ileum, jejunum, duodenum, and gastric outlet have also been reported [5,6].

Pre-operative diagnosis of gallstone ileus is difficult to make because of vague presentation, lack of specific diagnostic investigations and the rarity of disease. In 50% of the cases, diagnosis is made per-operatively [1,4,8]. The classical Rigler’s triad i.e. pneumobilia, intestinal obstruction, and, aberrant gallstone can only be found in 40–50 % of cases [1,4,8]. In our cases, we could not establish the diagnosis pre-operatively due to equivocal findings on investigations as reported in various studies [4,7]. Hence, the possibility of gallstone ileus needs to be considered not only in elderly patients but also in younger and middle aged patients presenting to surgical emergencies with intestinal obstruction even without the history of gallstones as seen in our case.

The standard surgical management of gallstone ileus is still a debate because of rarity of cases and lack of consensus guidelines. Various approaches to the management of gall stone ileus have been reported from minimally invasive shock-wave lithotripsy to major surgical exploration [[9], [10], [11]]. Surgical options include simple enterolithotomy, enterolithotomy and fistula closure with cholecystectomy, and bowel resection. Surgical decision is largely dictated by the condition of the patient. Enterolithotomy is usually considered in elderly patients with multiple comorbidities while enterolithotomy, fistula closure, and cholecystectomy is a safer approach in young, haemodynamically stable patients [11,12]. In our cases, we repaired the fistula in a single-stage procedure and our patient did well postoperatively despite presenting in shock. However, our patient was only controlled hypertensive with no other co-morbidities. His lack of multiple co-morbidities and relatively younger age might explain the uneventful postop course after single stage procedure.

Few studies advocate two stage procedures in all patients with gallstone ileus owing to increased morbidity and mortality of one stage procedure [10]. One Italian study reports significantly lower morbidity after conservative surgery compared to definitive surgery (67% vs 29%) [11]. However, our patient did well after definitive surgery despite presenting in shock. It gives an advantage of saving the patient from subjecting to general anaesthesia twice and also cuts down on the financial burden. Despite, the advantages of one-stage procedure, it is only appropriate in relatively healthy, younger patients with minimum co-morbidities. However, randomized controlled-trials need to be done to reach better conclusions and make proper decisions.

## 4 Conclusion

Gallstone ileus is a rare disease and surgical management strategy remains controversial. Randomised controlled trials need to be performed to draw definitive conclusions for the management of gallstone ileus. Further studies need to be done on comparison of spontaneous versus surgical closure of bilioentric fistulas to help surgeons make better choices.

## Conflict of interest

None.

## Sources of funding

This research did not receive any specific grant from funding agencies in the public, commercial, or not-for-profit sectors.

## Ethical approval

Ethical approval was sought from Institutional Review Board, Services Institute of Medical Sciences, Lahore, Pakistan.

## Consent

Written informed consent was obtained from the patient for publication of this case report and accompanying images. A copy of the written consent is available for review by the Editor-in-Chief of this journal on request.

## Author contribution

Nafeesah Fatimah: Involved in conception, design, and preparation of the manuscript, and final approval for publication.

Abubaker Shafiq Ahmed: Involved in conception, design and preparation of the manuscript.

Muhammad Umar Warraich: Involved in conception, design and preparation of the manuscript.

Usman Ismat Butt: Involved in conception, design and preparation of the manuscript.

Qamar Ashfaq Ahmad: Involved in conception, design and preparation of the manuscript.

Mahmood Ayyaz: Involved in conception, design and final approval of the manuscript.

## Registration of research studies

This is a case report not a research or a study, so Registration of Research Studies is not required.

## Guarantor

Nafeesah Fatimah and Mahmood Ayyaz.

## Provenance and peer review

Not commissioned, externally peer-reviewed.
